# Pulpless Teeth and Their Treatment

**Published:** 1887-11

**Authors:** George H. Cushing

**Affiliations:** Chicago.


ARTICLE II.

### PULPLESS TEETH AND THEIR TREATMENT.

BY GEORGE H. CUSHING, CHICAGO.

[Read before the Chicago Dental Society, May, 1887.]

In view of the fact that so much has recently been said and written upon the subject assigned to me for this evening, it would seem as though there could be no occasion for anything further being said relative to it now. But our daily experience shows us the importance of bringing it before our minds—again and yet again—with "line upon line and precept upon precept," in order if possible to secure a more universally successful practice than now prevails. There is hardly a day passes but that some of us witness the deplorable effect of ignorance or culpable negligence in this line of practice, and while this is so, it will be incumbent on us to discuss and re-discuss the subject till both ignorance and apathy concerning it are dispelled.

It is not proposed, nor would it be desirable here, to attempt an exhaustive study of the subject, and this paper will only endeavor to emphasize some of the more important points which should be observed in order to insure success, and to try and indicate the more frequent causes of failures in the treatment of pulpless teeth.

Pulpless teeth are not necessarily *dead* teeth. They are frequently so miscalled, however, and the miscalling leads to a misconception of their true conditions. The destruction and removal of the pulp of a tooth results primarily in simply depriving the dentine of its chief or perhaps only source of nourishment. It does not at all affect the integrity of the tooth as to its connection with the jaw, or as to the nourishment of the cementum whereby that integrity is maintained. If, then, after the pulp is destroyed and removed, the tooth can be put into such condition as not to provoke disease of the parts with which it is intimately associated, it may reasonably be expected that it will be retained as a useful member for very many years or even through a long life time. This being accepted as true, the first and most important inquiry is, what is the source of diseased conditions attending pulpless teeth, which do not show themselves in connection with those having living, healthy pulps? There can be but one answer to this question, viz: that septic matter contained within the pulp chamber or canals furnishes the sole irritating cause resulting in pericementitis, alveolar abscess, necrosis of the alveolar process, and sometimes of large areas of the maxillary bones. This can not be too strongly and persistently impressed upon the minds of the younger men (particularly) of the profession, though many of the older members would do well to give this fact greater consideration than they seem to do, for this is the very key-note of the problem of successful treatment.

Obviously, then, the first step in the treatment of such teeth is the removal from the pulp chamber and canals of all septic matter. This, of course, renders them aseptic, and of course employs in its accomplishment some of the various methods of disinfection.

To secure this condition, what then are the very important points to be considered? First, the isolation of the tooth to be treated, by means of the rubber dam or napkins. Second, the use of properly adapted instruments for the removal of the contents of the pulp chambers and canals, and last, but not least, the thorough disinfection of all instruments before using.

While it is possible that teeth treated with but slight regard to the above requirements may sometimes give but little trouble, yet the neglect of any one of these prime factors may render treatment tedious and difficult, which would have proved brief and simple had they been strictly observed, and such neglect may, and sometimes does, result in most disastrous consequences.

This aseptic condition having once been secured, the next consideration must be to render it permanent.

It is self-evident that if all septic matter is removed from the canals and pulp chamber, and they are then filled with an indestructible material which cannot be penetrated by moisture or by septic matter or gases, and does not carry contamination with or before it, the tooth must then indefinitely maintain its integrity as far as the conditions under consideration are concerned.

The point most important to be next considered is the introduction of the material with which the canals are to be permanently filled. Fortunately we have in gutta-percha a material that may be made to fill all canals with approximate perfection. This should be introduced with the greatest care so that the apical end of the roots may be perfectly closed. There should be no hurry about this part of the operation, but ample time should be taken to insure the desired result. The solution of gutta-percha in chloroform of the consistence of thick cream should be used at first and pumped in till there is assurance that it has reached the apical foramen. This should be done with smooth broaches adapted to the size of the canals. Gutta-percha cones should then be used where practicable, and they should be thoroughly packed. In very diminutive canals, or those of difficult access, small gold points filed to fit such canals (as suggested by Dr. Wassall) should be used; after the solution had been well worked in, they should be forced as far into the canals as possible without extending beyond the foramen. They act as wedges to force the gutta-percha to its place, and are useful where nothing else could be used.

The above briefly are the points to be kept prominent in our minds, and a faithful working out of details under them cannot fail of success.

Now a review of the most frequent causes of failure would seem to be unnecessary after having indicated the most important points to be observed essential to success, because it is obvious that a neglect of some one or all of the essentials claimed must be the cause of failure in almost all cases, yet it may be profitable to dwell upon them for a moment that we may be fortified against the temptations to careless methods which so often present themselves in the course of treatment of this class of cases.

The most potent cause of failure undoubtedly arises from a want of thorough comprehension of the first proposition laid down at the outset of this paper, viz.: that all these troubles arise from septic poisoning, and, in connection with that, of the failure to understand what disinfection really is. It seems to be impossible for some minds to grasp the idea that all the troubles which we so frequently see in connection with pulpless teeth can arise from a septic poison. The cause to such minds not being tangible is beyond their mental grasp as well, and while nominally accepting the theory, they say by their actions that they do not believe the cause to be as stated. Such a lack of confidence in the fundamental premise must of necessity beget carelessness in the methods pursued, which inevitably leads to bad results.

Again, the want of confidence in the premises here laid down is the result of neglect to study the subject by those who would fully comprehend it if they chose to apply their minds to it.

Indifference again as to the results comes sometimes from a feeling that the compensation to be received will not justify the time and trouble necessary to the accomplishment sought. Fortunately such ignoble motives are not often found actuating half-way reputable men.

But the chief causes of failure after these, are to be attributed to a lack of thoroughness in performing the details of the necessary operations. This will often be found to occur with those who are well posted, and who endeavor to do and think they are doing the very best that can be done. One man will fail to remove all the debris—while believing he has done so most thoroughly; another will remove most pefectly the contents of the canals, but fail to thoroughly disinfect them before filling; while still another may perform the first two steps in the most admirable manner and yet fail most sadly to properly fill the canals.

Men's minds are so differently, and I might say curiously, constituted that all these errors will sometimes eccur at the hands of men regarded as scientific, and who would be supposed to be thorough in all their methods.

A celebrated man, one who is high authority in the scientific world—and justly so, too—was observed to test the mouth with litmus paper which he held between his naked fingers. Conclusions drawn from such observations could hardly be valuable. If such men sometimes pursue unscientific methods, it is not to be wondered at that among those less highly educated such practices will occasionally occur.

This brief summary of the points essential to be observed to insure success on the one hand, and the dangers and errors to be avoided on the other, evidently can lead to but one conclusion, viz.: the importance of a thorough comprehension of what is needed to be done, and a practical training which shall evolve the skill necessary for its performance. This can come only from free discussion and clinical demonstration and practice.

It is hoped that the former may be induced by what has been suggested herein, and that such details as may be profitably considered here, and which were purposely avoided in the paper, will be introduced in the discussion which may follow; while the clinical teaching which should supplement the theoretical may be furnished hereafter, as it has been intimated that plans for a regular series of clinics are in contemplation.

Believing that this subject can not be too fiully discussed, and with the hope that what has been said may stimulate some at least to a more earnest study of this most important subject, the paper is left in your hands.—*The Dental Review.*

